# Association of pharmacogenomic, clinical and behavioural factors with oral levothyroxine (LT-4) dose of hypothyroid patients in Sri Lanka: a matched case control study

**DOI:** 10.1186/s12920-024-01849-z

**Published:** 2024-03-27

**Authors:** S. S. Dalugodage, Gayan Bowatte, Charles Antonypillai, S. Rajapakse, T. M. I. U. K. Tennakoon

**Affiliations:** 1 Department of Pharmacy, Faculty of Allied Health Sciences, University of Peradeniya, Peradeniya, Sri Lanka; 2 Department of Basic Sciences, Faculty of Allied Health Sciences, University of Peradeniya, Peradeniya, Sri Lanka; 3 National Hospital, Kandy, Sri Lanka; 4 Department of Molecular Biology and Biotechnology, Faculty of Science, University of Peradeniya, Peradeniya, Sri Lanka

**Keywords:** Hypothyroidism, Levothyroxine, Dose requirement, Single nucleotide polymorphisms (SNPs), Patient compliance, Comorbidities, Drug interactions

## Abstract

**Background:**

Hypothyroidism is a common endocrine disorder that exerts a substantial influence on people all over the world. Levothyroxine (LT-4) is the drug of choice for the treatment of hypothyroidism and the starting oral dose is typically ranging from 1.5 to 1.7 µg/kg/day. The target is to achieve an optimum serum TSH level of 0.4-4.0 mIU/L; hence, the dose is titrated accordingly. Once the LT-4 dose is adjusted to obtain the target TSH level, it usually remains stable for a long period of time in most cases. However, some of the patients require frequent dose adjustments and some of them require unusually high doses. Therefore, the aim of this study is to determine the association of pharmacogenomic, clinical and behavioural factors with the oral levothyroxine (LT-4) dose requirement of hypothyroid patients in Sri Lanka.

**Method:**

This study will be conducted as a matched case-control study and will involve primary hypothyroid patients who visit the diabetes and endocrinology clinic at the National Hospital, Kandy, Sri Lanka. We will recruit a total of 292 cases and select 292 controls from the clinic who are matched in terms of age, sex and Body Mass Index (BMI). An interviewer-administered questionnaire will be used to collect data from the participants (*n* = 584). Of the 584 patients, blood samples will be collected from a sub-sample (*n* = 150) for DNA extraction. Polymerase chain reaction-restriction fragment length polymorphism (PCR-RFLP) will be performed for single nucleotide polymorphisms (SNP) analysis.

**Discussion:**

Frequent dose adjustments of levothyroxine cause a serious economic burden to the healthcare system. By identifying the root causes of the variations in LT-4 dosage, a more comprehensive comprehension of hypothyroidism and its management can be attained in Sri Lanka. Furthermore, upon identification of a positive association/correlation between genetic polymorphisms and the LT-4 dose, SNP profiles can be used as a possible genetic marker for dose adjustment determination in future patients.

## Background

Thyroxine (T4) and triiodothyronine (T3) are the major hormones secreted by the human thyroid gland and they are collectively called thyroid hormones [1]. Thyrotropin releasing hormone (TRH), which is secreted by the hypothalamus, regulates the synthesis and secretion of thyroid stimulating hormone (TSH) from the anterior pituitary and TSH regulates the synthesis and secretion of thyroid hormones from the thyroid gland [2]. The thyroid gland produces a high amount of T4; the inactive form, while producing a small amount of T3; the active form. In peripheral tissues, inactive T4 converts to active T3 by the enzymatic activity of deiodinases. There are several isoforms of deiodinase enzymes, such as D1, D2 and D3. D2 is the main enzyme in activating the pro-hormone T4 into T3. Low levels of free serum T3 and T4 will increase TSH secretion via the negative feedback system [3]. Deficiency of thyroid hormones in the body causes hypothyroidism [3].

Hypothyroidism is a common endocrine disorder worldwide [4]. However, data on the prevalence of primary hypothyroidism in Sri Lanka is scarce. The prevalence of autoimmune thyroiditis in Sri Lanka has been found to be 16–20% [5]. It is characterized by high levels of serum TSH (reference range of serum TSH is 0·4–4·0 mIU/L) and there are several categories of the disease. Primary hypothyroidism is caused by a pathology in the thyroid gland itself, which results in reduced secretion of thyroid hormone. This results in higher TSH and normal or low thyroid hormone levels. Secondary hypothyroidism is caused by pathologies in the hypothalamao-pituitary unit. This results in low TSH and low thyroxine levels. Out of these two types of hypothyroidism, primary hypothyroidism is the most common type [6, 7]. Clinical (overt) hypothyroidism is manifested by an increased serum TSH level (> 10 mIU/L) and a reduced serum fT4 level (< 60 nmol/L), whereas sub-clinical hypothyroidism is manifested by a mildly increased serum TSH level (> 4 mIU/L) with a normal fT4 level [7]. However, the reference ranges may differ with the assay used, patient’s age, sex and ethnic group. Moreover, the upper limit of the adult serum TSH reference range typically increases with age [8].

Levothyroxine (LT-4) is the drug of choice for the treatment of hypothyroidism and it’s a synthetic T4 hormone [4]. The majority of the patients diagnosed with hypothyroidism require life-long treatment with levothyroxine [6]. Generally, patients with TSH levels > 10 mIU/L should start levothyroxine treatments [6, 9]. The starting oral dose of LT-4 is typically ranging from 1.5 to 1.7 µg/kg/day (equivalent to approximately 100–125 mcg/day). However, older patients or patients with coronary artery disease may receive a lower starting dose of levothyroxine (25–50 µg/day) [6, 10]. The LT-4 dose required by a patient can be determined by the total body weight, body mass index (BMI), lean body mass and ideal body weight using standard equations and the use of total body weight in this case may give the least accurate dose [11, 12]. The target is to achieve an optimum serum TSH level of 0.4-4.0 mIU/L and hence, the dose is titrated accordingly [3, 12]. LT-4 has a half-life of one week (about 7 days) and therefore serum TSH levels should be measured 4–6 weeks after starting the therapy or after a change in the dose. Once the target TSH level has been achieved, it can be reconfirmed after 3–6 months and then TSH monitoring should be done annually when stable [3, 6, 9, 13].

### Factors contributing to levothyroxine dose adjustments

Once the LT-4 dose is adjusted to obtain the target TSH level, it usually remains stable for a long period of time in most cases. However, around 10% of the patients, require dose adjustments during levothyroxine therapy and some of them will need unusually high doses of thyroid hormone replacement [14]. Factors including age, changes in body weight, coexisting medical conditions, concurrent medications, patient compliance, dietary habits, medication storage conditions and genetic factors may influence frequent dose changes of LT-4 [11, 13].

#### Coexisting medical conditions

Levothyroxine absorption is known to be affected by the gastric pH and hence, concomitant gastrointestinal (GI) diseases that impair gastric acid secretion or cause malabsorption may contribute to higher dose requirements [15, 16]. GI conditions such as *Helicobacter pylori* infection (reduce gastric acid secretion and produce ammonia), atrophic gastritis, coeliac disease, lactose intolerance, gastric bypass, inflammatory bowel disease and intestinal giardiasis are found to be associated with higher dose requirements [17, 18]. Patients with nephrotic syndrome may also require higher doses due to the urinary loss of thyroxine-binding globulin with thyroxine [19].

#### Concurrent medications

Some other medications, when taken along with LT-4 can lead to several drug-drug interactions and alter the LT-4 replacement dose requirements in patients. Proton-pump inhibitors (PPIs) including omeprazole, esomeprazole, lansoprazole etc., antacids and Histamine (H2)-receptor blockers reduce the gastric acid secretion and hence interfere with LT-4 absorption [14, 20]. In addition to that, ion exchange resins, bile acid sequestrants (cholestyramine, colestipol), sevelamer, laxatives, orlistat and sucralfate are known to reduce the absorption of LT-4 [20–22]. Moreover, supplements such as calcium and iron may form insoluble complexes with LT-4 inside the intestine and reduce the absorption [23]. Thus, concurrent use of these medicines may increase the dose requirements of LT-4 [14, 23]. Carbamazepine, hydantoins, phenobarbital and rifampicin may increase the hepatic metabolism of LT-4 and increase the dose requirement. Medicines such as amiodarone, iodide-including and iodine-containing radiographic contrast agents, methimazole, propylthiouracil and lithium may reduce thyroxine secretion and hence increase the dose requirement. Furthermore, oestrogen therapy, clofibrate, heroin/methadone, mitotane and tamoxifen may increase serum thyroxine binding globulin concentration and thus, they all increase the dose requirement of LT-4 [12, 14].

#### Patient compliance

Typically, in Sri Lankan health settings hypothyroid patients are advised to take their levothyroxine tablets with a full glass of water every morning, quickly after waking up. They are advised not to take any foods or beverages for about 30–60 min after taking LT-4. If the patient is taking medicines for another disease condition, he/she is informed to separate LT-4 and those medications by 2–4 h. Furthermore, patients are encouraged to take the same brand name product of LT-4 [24, 25]. Poor compliance with the therapy is a major factor to consider when finding causes for frequent dose adjustments or higher dose requirements.

A recent study has concluded that taking LT-4 half an hour before breakfast, an hour before the main meal of the day and at bedtime (minimally 2 h after dinner) are equally effective and can be used to enhance patient compliance [26].

#### Dietary habits

Frequent consumption (more than twice a week) of soy-based products (soybeans, soy milk, tofu), foods high in fiber (bran flakes, fiber drinks, broccoli, nuts, whole grains), foods high in iodine (cod, plain yogurt), grapefruit/grapefruit juice and coffee/tea with LT-4 may increase the serum TSH levels and may increase LT-4 dose requirements [20, 23].

#### Medication storage conditions

According to the guidelines, levothyroxine should be stored at 20–25 °C (68–77 °F) and protected from light and moisture [25]. A recent study has proven that improper storage conditions can increase serum TSH levels and thereby increase the dose requirements [27].

#### Genetic variations

Genetic polymorphisms are known to be responsible for most of the common genetic variations among humans [28]. Researchers suggest that the wide variability of LT-4 doses required by the patients may have an association with SNPs of TSHRs, THRs, thyroid hormone transporters and deiodinase enzymes [14, 28, 29]. A study conducted in 2013 has concluded that single nucleotide polymorphisms (SNPs) rs225011, rs7140952, rs225012 and rs2839858 in DIO2 gene are not correlated with the replacement doses of the LT-4 whereas, rs7140952 polymorphism was found to be associated with components of metabolic syndrome including blood pressure and central obesity. Moreover, the researchers have stated that this association might be population dependant [11]. In another study, an association between TSHR gene SNP (rs2268458), which is in intron 1, and a higher incidence of Graves’ disease has been found [30]. Furthermore, the CC genotype of TSHR SNP (rs2239610) has been found to be associated with higher serum concentrations of fT4 [31]. Torlontano et al. have found a positive association between DIO2 polymorphism (D2 Thr92Ala) and the required dose of L-T4 in thyroidectomised patients [32].

According to the best of our knowledge, no studies have been conducted to explore the association between above mentioned factors and LT-4 dose adjustments in Sri Lankan population. Therefore, we intend to investigate the possible association of LT-4 dose requirement in hypothyroid patients and pharmacogenomic, clinical and behavioural factors.

### Research objectives

The goal of this study is to determine the association of pharmacogenomic, clinical and behavioural factors with oral levothyroxine (LT-4) replacement dose of hypothyroid patients in Sri Lanka. Specifically, we are going to determine;

- The association of factors including coexisting medical conditions, concurrent medications, patient compliance, dietary habits and medication storage conditions with the LT-4 replacement dose.
- The association between genetic polymorphisms in the THR gene, TSHR gene and DIO2 gene and the LT-4 replacement dose.

## Methods/design

### Study design

A matched case-control study will be conducted, with the selection of hypothyroid patients who are currently receiving LT-4 treatment as study participants.

#### Definition of the cases

Patients visiting the Kandy National Hospital’s diabetes and endocrinology clinic with a LT-4 replacement dose > 1.7 µg/kg/day.

#### Definition of controls

Patients visiting the Kandy National Hospital’s diabetes and endocrinology clinic with a LT-4 replacement dose 1.7 µg/kg/day or less (normal doses).

These patients will be identified as hypothyroid patients by the investigation of a physician and laboratory tests (TSH and fT4). Of the pool of hypothyroid patients with normal doses (1.7 µg/kg/day or less), the controls will be selected using convenience sampling. Age, gender, and BMI will be matched in cases and controls. One control per case will be selected.

### Study setting and population

Primary hypothyroid patients who visit the diabetes and endocrinology clinic at the National Hospital, Kandy, Sri Lanka will be recruited (*n* = 584). Sample size was calculated using following formula [33].

$$ n\left(each\,group\right)=\frac{\left({p}_{0}{q}_{0}+{p}_{1}{q}_{1}\right){\left({z}_{1-\alpha /2}+{z}_{1-\beta }\right)}^{2}}{{\left({p}_{1}-{p}_{0}\right)}^{2}}$$

Whereas;

p_0_ = Proportion of controls with exposure (Nair S, et al.) [34].

p_1_ = Proportion of cases with exposure (Nair S, et al.) [34].

z _1−α/2_ = Standard normal variate for level of significance.

z _1−β_ = Standard normal variate for power.

q_0_ = 1-p_0_.

q_1_ = 1-p_1_.

We will recruit a total of 292 cases and select 292 controls from the clinic who are matched in terms of age, sex and BMI.

#### Inclusion criteria: cases

1. Patients with primary hypothyroidism who require higher doses of thyroxine (more than 1.7 µg/kg/day) for the preceding 3 months.
2. Age 18 years to 65 years.
3. Patients who have given consent.

#### Inclusion criteria: controls

1. Patients with primary hypothyroidism requiring thyroxine normal doses (1.7 µg/kg/d or less) for the preceding 3 months with normal TSH.
2. Age 18 years to 65 years.
3. Patients who have given consent.

*Exclusion criteria: Cases and controls*.

1. Patients who are pregnant or planning for pregnancy within 6 months.
2. Patients with secondary hypothyroidism.
3. Patients who are newly diagnosed with hypothyroidism (< 1 year).
4. Patients with a history of differentiated thyroid malignancies.
5. Patients who are being treated for psychiatric illness.
6. Patients with established atherosclerotic cardiovascular disease or arrhythmias.

### Sample and data collection

Cases and controls will be selected using convenience sampling. Informed written consent will be obtained from all the participants. A researcher-administered questionnaire will be administered to obtain data on age, gender, ethnicity, year of diagnosis, aetiology of hypothyroidism, the current dose of thyroxine and brand, last TSH within 3 months, time of thyroxine ingestion and time to next meal or drink, dietary habits, number of days missing the tablets per month, whether the patient is storing the tablets correctly, interfering drugs and the other drugs that the patient is on and the other medical conditions that the patient is having. The body weight and height of the patient will be measured using the same scale for every patient, and BMI will be calculated.

### Blood sample collection

A sub-sample of cases (*n* = 75) and their respective controls (*n* = 75) will be recruited randomly from the initial sample of 584 patients for SNP analysis due to the limited availability of resources. Blood samples will be collected from those patients (*n* = 150) and will be used for SNP analysis by PCR/RFLP method.

### PCR-RFLP

The current study will investigate the possible association between LT-4 doses in hypothyroid patients and THRα rs939348 SNP, TSHR rs2268458 SNP, TSHR rs2239610 SNP and rs225011, rs7140952, rs225012 and rs2839858 SNP’s in DIO2 gene. The PCR primers, restriction enzymes and the expected sizes of the amplified and digested fragments of the examined SNPs are shown in Tables 1 and 2.

**Table 1:** PCR primers, restriction enzymes, and the expected sizes of the amplified and digested fragments of the SNPs associated with TSHR and THRα genes [35]

SNP	PCR product (bp)	Primers	Restriction enzyme	Restricted fragments (bp)
Rs939348	190	F: 5’-CCTGTGTCTCCCAGCTTAGG-3’ R: 5’-CCACCAGACTCACAGCCTCT-3’	MesI	C allele190 T allele48 & 142
Rs2268458	162	F: 5’-CTAACCAGCAGAGGGAGCAC-3’ R: 5’-CCACTGCTTAAAGCCCAGAT-3’	AluI	T allele162 C allele 100 & 62
Rs2239610	172	F: 5’-CCAGAGATCAAGGGCATCTG-3’ R: 5’-CCAAGTGTGGGCGATTAAGT-3’	NlaIV	G allele172 C allele142 & 30

**Table 2:** PCR primers, restriction enzymes and the expected size of the amplified and digested fragments of the SNPs associated with DIO2 gene [11]

SNP	PCR product (bp)	Primers	Restriction enzyme	Restricted fragments (bp)
rs225011	323	F: 5’-CAGCTGCAGAATTCTTTCCA-3’ R: 5’-GGCCCCAGGATTCATATTTC-3’	XmaJI	TT: 323 CC: 203/120
rs225012	335	F: 5’-TGGAGGCTTCTACTGCC ACT-3’ R: 5’-AGGCTGCAAACTTTCCTTCA-3’	Eam1104I	AA: 335 GG: 243/92
rs7140952	303	F: 5’-CCAAAAGAAACCCCTCACAA-3’ R: 5’-GGCAGGTGGAGAGAGTGGTA-3’	BfmI	TT: 303 CC: 101/202
rs2839858	370	F: 5’-TTACCTGCCATCATGCC TCT-3’ R: 5’-GCTCGTGAAAGGAGGTCAAG-3’	MnlI	AA:158/95/41/46/26/4 CC:204/95/41/46/26/4

The PCR will be carried out using a blood PCR Kit. The genomic DNA will be amplified using a PCR programme that is suitable for the above mentioned SNPs. Tables 1 and 2 summarizes restriction enzymes and restricted fragments for all SNPs. The PCR products and digested fragments will be detected using electrophoresis on 2% agarose.

### Gene sequencing to identify new SNP variants

Direct Sanger sequencing will be done to identify new variants of DIO2 genes in patients who have been on thyroxine more than 2.5 micrograms/kg/day dose for the last 3 months period. The obtained gene sequences will be compared with the reference sequence from the National Centre for Biotechnology Information (NCBI).

### Statistical analysis

Before data entry, all data forms and questionnaires will undergo a thorough check for errors, and necessary corrections will be made. The data will be entered using a data entry program equipped with built-in range and consistency checks. An analysis of frequency distributions will be conducted to identify any outliers.

Data will be analysed using Stata software (Stata Corp. 2021. Stata Statistical Software: Release 17. College Station, TX: StataCorp LLC.). The mean and standard error will be presented for data that follows a normal distribution. Conversely, non-normal distributed data will be expressed as medians with interquartile ranges. Descriptive analysis of all variables and comparisons between case and control groups will be presented. T-tests will be used for continuous variables, and Chi-square tests for discrete variables. Logistic regression and multinomial logistic regressions will be used to investigate the associations between predictor variables, such as coexisting medical conditions, concurrent medications, patient compliance, dietary habits and medication storage conditions with the LT-4 replacement dose. Potential confounders will be identified using Direct Acyclic Graphs (DAG) and existing literature. The identified confounders will be adjusted in the multivariate models. All the results will be presented before and after adjustment for confounding variables. The genotype distributions of SNPs will be analysed in agreement with the Hardy-Weinberg equilibrium. The association between genetic polymorphisms and the LT-4 replacement dose will be analysed using logistic and multinomial logistic regression models adjusting for potential confounders.

In gene sequencing, the obtained gene sequences will be compared with the reference sequence from the National Centre for Biotechnology Information (NCBI). The sequences will be analysed using BioEdit software.

## Discussion

Hypothyroidism is a common endocrine disorder worldwide [4]. Females are more prone to the disease than males and the prevalence increases with age [3, 8]. If hypothyroidism is left untreated, it may cause severe adverse effects and ultimately death [8]. Hypothyroidism can be diagnosed and monitored by evaluating clinical symptoms and thyroid function testing; mainly serum TSH level and serum fT4 level [9]. Hypothyroidism shows symptoms such as mild to moderate weight gain, hyperlipidaemia, skin manifestations/dry skin, fatigue, constipation, coarseness or loss of hair, bradycardia, hypothermia, myalgia, depression, menstrual irregularities and lack of concentration [3, 11]. However, clinical manifestations of hypothyroidism may range from life threatening to no signs or symptoms [8].

The common causes for primary hypothyroidism are autoimmune disease of the thyroid gland/Hashimoto thyroiditis (major cause), thyroid surgery/surgical removal of the thyroid gland, thyroid gland ablation with radioiodine or external radiation when there is an iodine sufficiency [6, 7]. There are several other causes for primary hypothyroidism and they can be listed as antithyroid medications (propylthiuracil), thyroid gland tumors, biosynthetic defects in iodine organification, iodine deficiency or excess (Wolff-Chaikoff effect) and some medications such as amiodarone (contains iodine), lithium, tyrosine kinase inhibitors and cytokines (interferon-γ and interleukin-2) [3, 6].

Generally, hypothyroidism can be adequately treated with a consistent daily dose of oral levothyroxine. However, around 10% of the patients, require frequent dose adjustments during levothyroxine therapy and some of them will need unusually high doses of thyroid hormone replacement [14]. Patients who undergo multiple levothyroxine dose adjustments consume more healthcare resources and hence, these frequent dose adjustments cause a serious economic burden to the healthcare system [36]. Although many studies have been conducted to explore the association of levothyroxine dose adjustment with demographic factors and genetic factors, there are no studies available on the Sri Lankan population. Especially, genetic factors may differ from one population to another. Authors of previous studies that have been conducted to explore the possible associations between SNPs and LT-4 replacement dose, have declared that their results might be population dependant [11, 32]. Therefore, it is important to see the applicability of these already-described factors to our population.

Understanding underlying causes for variations in LT-4 dose will lead to a better understanding of hypothyroidism and its management in Sri Lanka and similar low-resource settings. Furthermore, if a positive association/correlation is found between genetic polymorphisms and the LT-4 dose, SNP profiles can be used as a possible genetic marker for dose adjustment determination in future patients. Moreover, the results of this study will guide to launch of further research studies in this field.

A potential limitation of this study is that our sample for the SNP analysis is small due to the limited availability of resources. We expect this study will help to understand the causes of frequent dose adjustments in LT-4 treatment.

## Data Availability

Not applicable.

## Abbreviations

BMI: Body mass index
D1, D2 and D3: Deiodinases 1, 2 and 3
DIO2: Deiodinases 2 gene
fT4: Free thyroxine
GI: Gastrointestinal
LT-4: Levothyroxine
NCBI: National Centre for Biotechnology Information
PCR: Polymerase Chain Reaction
PPI: Proton-pump inhibitor
SNP: Single nucleotide polymorphism
SPSS: Statistical analysis for the social sciences
THR: Thyroid hormone receptor
TSH: Thyroid stimulating hormone
TSHR: Thyroid stimulating hormone receptors
TRH: Thyrotropin releasing hormone
T4: Thyroxine
T3: Triiodothyronine
